# The Soul of Octogenarian BJORL

**DOI:** 10.5935/1808-8694.20130096

**Published:** 2015-10-08

**Authors:** Silvio Caldas Neto

**Affiliations:** Adjunct Professor and Head of the Department of Otorhinolaryngology - Federal University of Pernambuco (UFPE). Senior Associate Professor of Otorhinolaryngology - Medical School of the University of São Paulo (FMUSP).

Few people had the privilege to head and thus become deeply acquainted with the largest and longest serving national scientific publication in the field of Otorhinolaryngology in Brazil. I am proud to have been one of them, in the capacity of Director of Publications for the ABORL-CCF between 2005 and 2010; and I admit that I felt this nostalgia when I was invited to write this editorial. I was suddenly taken by that sense of responsibility towards my colleagues, of representing them in a distinct and correct way. And I felt it could not be different now, in this mission to convey to them my view on the scientific importance of the BJORL in its 80 years of life, or at least in the meager five years within which I took part in its history. I searched and gathered data, made statistical calculations and correlations and strived to give scientific evidence to my words, as if thereby I were honoring the fundamental principles that govern and that have always ruled our journal - scientific excellence and editorial sophistication. Suddenly, I started to wonder if just the opposite was about to happen, if I was using this editorial space to pay homage to myself, because of half a decade within eighty years; as if the privilege had belonged to the journal, and not to its director. I then decided to put aside accomplishments, achievements, improvements, or other parameters that could attempt to testify for an intended management efficiency, and decided that I should rather speak, if possible, from the soul of our journal, which perhaps portrays more for the little things of day-to-day than the charts.

My memory swung back and forth with several small facts and situations which, for me, define this very soul, for example, the care of our Assistant Director at the time - Regina Helena, in the correction of minor editorial errors, numbering tables and figures and even (phew!) references! It also came to my mind a most noble João Mello, snapped to his principles, refusing a decision he deemed not fair, even though such an attitude would not be known to most people, but only a few supporters and some many critics… I remembered the recent stalemate between privileging the papers from otolaryngologists, who were, after all, the owners of the journal and to whom we owed accountability, or give equal treatment to papers from other professionals, often of great value to increase our impact factor - should we privilege the ENTs or the journal? We have always taken pride in having the decision made by our consciousness, regardless of what it was. We also had the endless discussions about the effects of the political classification of scientific journals and graduate programs made by CAPES on national medical journals… attempts to clarify to colleagues the nuances behind the new classification… the serenity of all vis-à-vis the criticisms and fears arising from misinformation on this subject… the painful decision to discontinue the Revista Brasileira de Otorrinolaringologia and invest in the Brazilian Journal of Otorhinolaryngology… and the very difficulty (thankfully solved with patience and creativity) to make these changes happen without much suffering and without changing the routine of the journal readers.

I apologize for expressing such personal feelings, but I must say that it also came from the broth of my memories, the look of pride of a father who saw himself continued in his offspring, greater encouragement, perhaps, for a soul tormented by our apparent finitude. Professor Nelson Caldas, my mentor and of so many others around the country, would also celebrate eighty years this year and I cannot help but make a connection between his personal and professional life and the history of the journal and that of the very Brazilian Otolaryngology. The spirit with which he branded his entire career is the same fashion that has been driving the course of our journal, based on scientific and ethical rigor, and in constant pursuit of self-improvement. A spirit aware that the scientist is one who constantly seeks the truth, not one who has found it. And, as he certainly saw continuity in me and in all his disciples, we also see ourselves continued in that little mark that we left in the history of the Brazilian Journal, to which we should be thankful for.

Finally, it came to my mind the bimonthly meetings of our Board of Publications along with the entire ABORL-CCF Executive Board… and the passionate involvement of all vis-à-vis issues related to the journal, showing that it truly was the darling of Otolaryngology. The support of the presidents, the search for sponsorship, the small ideas that could make big differences, the spirit of all devoted to the institutional interest above any other… This is the soul of our BJORL and it is to such soul that I should pay tribute in this festive year, an homage that I also pay to all of those who have helped and still help on this success story, either as founder, as director, researcher, translator, secretary, organizer, sponsor or many other functions involved in this magnificent job that has dignified our specialty and that was, after all, recognized by the high level indexing agencies in the world with our most recent ranking in the Journal Citation Reports from Thomson Reuters, a long dream cherished by us all and for which we have given ourselves as much as we could.

Congratulations, BJORL! Best wishes! Many years of life!

